# Robust, versatile DNA FISH probes for chromosome-specific repeats in *Caenorhabditis elegans* and *Pristionchus pacificus*

**DOI:** 10.1093/g3journal/jkac121

**Published:** 2022-05-14

**Authors:** Renzo S Adilardi, Abby F Dernburg

**Affiliations:** Department of Molecular and Cell Biology, University of California, Berkeley, Berkeley, CA 94720-3220, USA; Howard Hughes Medical Institute, Chevy Chase, MD 20815, USA; Department of Molecular and Cell Biology, University of California, Berkeley, Berkeley, CA 94720-3220, USA; Howard Hughes Medical Institute, Chevy Chase, MD 20815, USA; Biological Systems and Engineering Division, Lawrence Berkeley National Laboratory, Berkeley, CA 94720, USA; California Institute for Quantitative Biosciences, Berkeley, CA 94720, USA

**Keywords:** in situ hybridization, tandem repeats, karyotype, *Caenorhabditis elegans*, *Pristionchus pacificus*

## Abstract

Repetitive DNA sequences are useful targets for chromosomal fluorescence in situ hybridization. We analyzed recent genome assemblies of *Caenorhabditis elegans* and *Pristionchus pacificus* to identify tandem repeats with a unique genomic localization. Based on these findings, we designed and validated sets of oligonucleotide probes for each species targeting at least 1 locus per chromosome. These probes yielded reliable fluorescent signals in different tissues and can easily be combined with the immunolocalization of cellular proteins. Synthesis and labeling of these probes are highly cost-effective and require no hands-on labor. The methods presented here can be easily applied in other model and nonmodel organisms with a sequenced genome.

## Introduction

Fluorescence in situ hybridization (FISH) targeting chromosomal DNA has long been a powerful cytological tool to study chromosomes and nuclear architecture in fixed samples (Pardue and Gall 1969; Bauman *et al.* 1980). Successful application of this technique depends on design and synthesis of suitable labeled probes, combined with development of fixation and hybridization methods appropriate to address specific biological questions.

While many experiments require custom-designed probes to specific genomic regions of interest, a wide range of questions can also be addressed using probes to tandemly repeated sequences. Such repeats are often a major component of eukaryotic genomes and are typically concentrated at telomeric and pericentromeric regions. Multigene families such as ribosomal RNA genes (rDNA) and tandem repeats of varying lengths (satellite DNA) are commonly used as FISH targets in studies of genome organization, karyotype evolution, or chromosome segregation (e.g. Dernburg *et al.* 1996; Nguyen *et al.* 2010; Heckmann *et al.* 2013; Ruiz-Ruano *et al.* 2016). These and other repetitive sequences that are restricted to one or a few genomic loci are particularly valuable for cytological studies.

Several technical features make tandemly repeated sequences favorable targets for DNA FISH experiments. Probes that detect such sequences are easy to generate, since one or a few short, synthetic oligonucleotides can hybridize to many clustered targets. Short, synthetic DNA fragments are ideal FISH probes, particularly for whole-mount samples, as they readily diffuse through tissue fixed with moderate concentrations of formaldehyde (i.e. 2–5%), which is sufficient to preserve morphology during the denaturation steps required for DNA FISH, usually accomplished with a combination of heat and formamide. DNA oligonucleotides can readily be labeled with fluorophores or haptens either during chemical synthesis or afterward, using enzymatic methods. Synthesis of probes with modified backbones, such as locked nucleic acid or peptide nucleic acid chemistries, can further enhance detection by stabilizing probe-target hybrid molecules. Detection of high copy number sequences is inherently robust due to favorable hybridization kinetics arising from the high effective concentration of the target. Thus, repetitive probes typically produce more intense and reliable signals than probes targeting single-copy sequences spanning similar genomic intervals. This is particularly advantageous in well-preserved whole-mount cells or tissues, where hybridization of the probe to the target competes with reannealing of denatured chromosomal DNA during the hybridization process. While synthetic oligonucleotide probes can also be designed to detect single-copy sequences (e.g. Oligopaints; Beliveau *et al.* 2012), they must be used at a much higher total concentration, at least proportional to the sequence complexity. This requires significant effort and cost to amplify complex probes from synthetic libraries, which typically yield only low femtomolar quantities of each oligonucleotide (Schmidt *et al.* 2015).

The genomes of *Caenorhabditis elegans* and most other sequenced nematodes are compact and have low repeat content compared to many other eukaryotes (Rödelsperger *et al.* 2013). Consequently, only a few repetitive FISH probes have been described for *C. elegans*: these include probes against the telomere repeats (TTAGGC)n, the large ribosomal RNA gene cluster (8S, 5.8S, and 26S rDNA) on chromosome I, the tandemly repeated 5S rDNA locus on chromosome V, and 2 clustered repeats on the X chromosome (Albertson 1984; Dernburg *et al.* 1998; Phillips *et al.* 2005; Ferreira *et al.* 2013).

A new reference *C. elegans* genome was recently assembled based on long-read sequence data for the VC2010 strain (derived from the N2 Bristol strain), which increased the accuracy of repeat annotations (Yoshimura *et al.* 2019). This study reported an additional 1.8 Mb of tandem repeats and other duplications relative to previous assemblies, a significant difference in a genome comprising ∼100 Mb. Similar telomere-to-telomere genome assemblies with accurately annotated repetitive sequences are becoming the norm for many nontraditional model species, as well as for specific isolates or strains of highly studied organisms.

Leveraging current genome assemblies, we searched the genomes of *C. elegans* and its distant relative, *Pristionchus pacificus*, for tandem repeats suitable for DNA FISH in whole-mount tissues. We selected candidate repeats restricted to a single major genomic locus, designed simple, inexpensive FISH probes, and tested their performance in different tissues. Through this straightforward approach, we have designed and validated a set of probes that include at least 1 locus per chromosome for each species. The criteria we used proved to be highly reliable for target selection and probe design and can be easily extended to design FISH probes for other model and nonmodel organisms.

## Materials and methods

### Tandem repeat analysis

Chromosome-level genome assemblies from *C. elegans* (“VC2010,” Yoshimura *et al.* 2019), and *P. pacificus* (“El Paco,” Rödelsperger *et al.* 2017) were analyzed using Tandem Repeat Finder v4.09 (Benson 1999). A maximum periodicity of 200 bp and default parameters were used (match = 2, mismatch = 7, indels = 7, minimum alignment score to report repeat = 50). The total span of each repeat was calculated using the genomic indices. The output data for each chromosome were filtered to select repeats spanning more than 5 kb.

The candidate repeats were then analyzed using the online BLASTN tools from wormbase.org against the *C. elegans* “VC2010” assembly and from pristionchus.org against the *P. pacificus* “El Paco” assembly to characterize their genomic distribution. Default parameters (blastn matrix: 1–3; gap penalties: existence: 5, extension: 2) and an *E*-value threshold of 1E + 1 were used in both cases. Based on the BLASTN results, candidate repeats were analyzed further if they met 2 conditions: (1) The hits were clustered at only 1 major chromosomal locus and (2) any hits on other chromosomes were not clustered. In cases where portions of sequence motifs showed hits on other loci, candidate sequences were further trimmed and reanalyzed to eliminate potential cross-hybridization. To generate oligonucleotide probes ≤30 bp in length, longer motifs were split into nonoverlapping subsequences of approximately 20 bp, each of which was reanalyzed by BLASTN, as described above, to confirm their unique localization. A summary of BLASTN results with the number of perfect and imperfect hits for each oligonucleotide sequence is presented in Supplementary Tables 1 and 2.

Potential intra- and intermolecular hybridizations of each potential probe sequence were also evaluated using the Multiple Primer Analyzer web tool from Thermo Fisher (sensitivity for dimer detection = 3; Supplementary Note 1). Although this analysis revealed potential for some of the probes to form homo- or heterodimers, this did not compromise their performance. The resulting candidate probes, consisting of 1 or 2 oligonucleotides per repeat, were analyzed experimentally for specific and robust hybridization (Tables 1 and 2).

**Table 1.** List of locus-specific oligonucleotide FISH probes for *C. elegans*.

Chr	Probe name	Repeat position	Span (kb)	Oligonucleotide probe sequences (2 ^nd^ oligo in italics)	Length (b)	GC%	T_m_	Complete tandem repeat motif (probes in bold)	Length (b)
I	I-1	10374806 - 10387363	12.58	**TCTTTCTGAAATTCTAAGAA**	20	25	22.2	TTTTGGTAAAAGAAAACCATTGTCAACTGAATAGGTTGATTTGTGTTT**TCTTTCTGAAATTCTAAGAA**	68
	I-2	14697045-14704558	7.51	**AATTTTCACTTTCGGTAAAT**	20	25	24.2	TGCCGATTTGCCGGA**AATTTTCACTTTCGGTAAAT**	35
II	II-1	6603736-6620597	16.86	**CGAGATGATCGGTCCAGAATACAGC**	25	52	38.4	CAGTATTTTGGGGTCTCTCCCTAGTTGTTAGGTAACTTTATACTTTTCTTCTTCTATTTCTT**CGAGATGATCGGTCCAGAATACAGC**	87
III	III-1	9125488-9139755	14.27	**CAGTTGAGACTACACCATATACCGG**	25	48	35.9	AGGATCAACAGCTTCTCCACCAACTGGAACCACCGATGAGCCTGGATCTT**CAGTTGAGACTACACCATATACCGG**TGAGACTCCATCAGTACCTAC	96
IV	IV-1	1966969-1972014	5.05	**CCGTAAATCTACAGTAATACC**	21	38.1	26.1	**CCGTAAATCTACAGTAATACC**	21
	IV-2	3243704-3271880	28.18	**TCACTCAAAATCCTGAGCC**	19	47.4	30.0	**TCACTCAAAATCCTGAGCC**	19
	IV-3	12787278-12803238	15.96	**CTTCTGGTAATGTTCCCATAATTGG,*CTCATAAGTAACTAGTATGGGAC***	25, 23	40, 39.1	33.4, 28.5	**CTTCTGGTAATGTTCCCATAATTGG**GTTAAATACCA***CTCATAAGTAACTAGTATGGGAC***TGAAAAGATACTAAATGAGCTTATTCTAAGGGTGAAG	96
	IV-4	14987799- 15001823	14.02	**CAGTTCATAAGGGGGACCTT**	20	50	31.9	ATCCTTTGGAGCTGAAGATT**CAGTTCATAAGGGGGACCTT**GTG	43
V	V-1	6165504-6179481	13.98	**CCTCCTGTTTCAGTTTATCATCCT,*TCTCCTTTTTCAGTTTAGCATCAG***	24, 24	41.7, 37.5	33.7, 32.6	**CCTCCTGTTTCAGTTTATCATCCT*TCTCCTTTTTCAGTTTAGCATCAG***	48
	V-2	8761483- 8777144	15.66	**CTCGTTATGTCGGTTGAAGACACAATTGGA**	30	43.3	40.9	GTGGTGCTTGAGGAGCTGGATTCAGTTGAG**CTCGTTATGTCGGTTGAAGACACAATTGGA**	60
	V-3	14861885-14869289	7.4	**GATATCGTAGCGTTTTTTGGTG**	22	40.9	31.2	**GATATCGTAGCGTTTTTTGGTG**GAATATGGAAAAATAAAAAAGTGCTAC	49
X	X-1	7586210-7592860	6.65	**CGCCGGTTTCGCTTTGAGCG**	20	65	40.4	**CGCCGGTTTCGCTTTGAGCG**ATTCCTTACCCTTAAATGGG	40
	X-2	17825386-17836686	11.30	**CACTTCGACTCCATCCACCAGC**	22	59.9	38.5	**CACTTCGACTCCATCCACCAGC**ACTGCTTCGAGTACGACAGAAAG	45

**Table 2.** List of locus-specific oligonucleotide FISH probes for *P. pacificus*.

Chr	Probe name	Repeat position	Span (kb)	Oligonucleotide probes (2 ^nd^ oligo in italics)	Length (b)	GC%	T_m_	Complete tandem repeat motif (probes in bold)	Length (b)
I	I-1	8491799-8511776/8551777 - 8558864	27.06	**GCCTTGAGCTTCGCCTGTTCTTCGG**	25	60	43.5	GGCCTTCTTCTCTTCTTCA**GCCTTGAGCTTCGCCTGTTCTTCGG**CGGTCTTCTTGGCATCCTT	63
	I-2	15772551 - 15784070/15789511-15807243/15847728-15890937	72.46	**ACCTCGTGGAGTCCATT**	17	52.9	29.6	**ACCTCGTGGAGTCCATT**CGATCATTGCTCTGCTCCACTTCAAGTCATAATCATTG	55
II	II-1	5022684-5059631	36.95	**GGGAGGGTAGACAGTTTACCCACACCAGAA**	30	53.3	44.4	GGGGA**GGGAGGGTAGACAGTTTACCCACACCAGAA**ACCTCAAGATCTTCGACGAATGGGTTTCCTGGAAAGTTGGCTACATTGAG	85
III	III-1	5716734-5757229/5760170 - 5762654	43.32	**CGTTGACATTGCACGATCGAATTCC**	25	48	38.6	TT**CGTTGACATTGCACGATCGAATTCC**GCAAGGAGAGAC	39
IV	IV-1	11295637-11335802	40.17	**TCATTGAAATGATCACAATCATTGA**	25	28	31.3	ATGA**TCATTGAAATGATCACAATCATTGA**G	30
	IV-2	30637612 – 30681023	43.41	**CTGATGCGTTCTCTACATTTTCGCC**	25	48	37.9	ACTCAAAATGTCAATGAAATTGGCTAAAAAAATGAAAAGTTGAACTTAGATTTCTCACGCAAAGGACATTGCTTTTCATTATTAAATTAATGATTA**CTGATGCGTTCTCTACATTTTCGCC**TAGCCTATGGTTGTGCAAGTGCACTCGTTCACAGAGGAC	160
V	V-1	18957565-18980331	22.77	**GACACTGGCGGTGTTCATTGAGAAC**	25	52	39.8	TTTTTGATTGATTGTTAGATTGAGAATGTGAGGCCCATGCAGATAACTATAGATCCATGCATGCAGGATCTTACGATGA**GACACTGGCGGTGTTCATTGAGAAC**	104
X	X-1	1-29285/69286 - 80443	40.44	**GGTGGTCGACGGCTGCGTCG**	20	75	43.4	**GGTGGTCGACGGCTGCGTCG**ACTGAAGAGT	30
	X-2	9167758-9180432/9222015-9236383	27.04	**TCCGGGGCTTTAGATGAGTTAGA,*GGAGAAACGATCGAGTTGTATATC***	23, 24	47.8, 41.7	36.2, 32.3	AAAGAGATACTGAAATAATCAT**TCCGGGGCTTTAGATGAGTTAGA**GGGGGGGGG***GGAGAAACGATCGAGTTGTATATC***TTT	81
	X-3	15501721-15528787	27.07	**TCCCTTTGTTCCGCAGTCCG**	20	60	38	TCATCATGAATGGGATTACGGTA**TCCCTTTGTTCCGCAGTCCG**	43

We also calculated melting temperatures (T_m_) for the selected oligos using the Biopython “Bio.SeqUtils.MeltingTemp” module (Cock *et al.* 2009), as in Beliveau *et al.* (2018), which is based on a nearest-neighbor thermodynamics method from Allawi and SantaLucia (1997). Default parameters and corrections for the hybridization conditions (585 mM [Na+] and 48% formamide, see FISH protocol below) were applied (Tables 1, 2, and Supplementary Table 3). Although these predicted T_m_ values varied widely, we found that in practice, all probes hybridized robustly and specifically at a standard annealing temperature of 37°C. This agrees with previous work using probes against *Drosophila* satellite repeats that vary markedly in GC content and predicted T_m_ values (e.g. Dernburg *et al.* 1996).

### Oligonucleotide probes

Probes were ordered from IDT as single-stranded DNA oligonucleotides labeled with fluorophores at their 3′ ends (100 nM, HPLC purification). To combine different probes in the same experiment, oligos were labeled with Cy3, Cy5, 6-FAM (6-Carboxyfluorescein), or Alexa Fluor 488. Lyophilized oligos were resuspended at 100 μM in MilliQ water, aliquoted, and stored at -30°C.

**Fig. 1.**
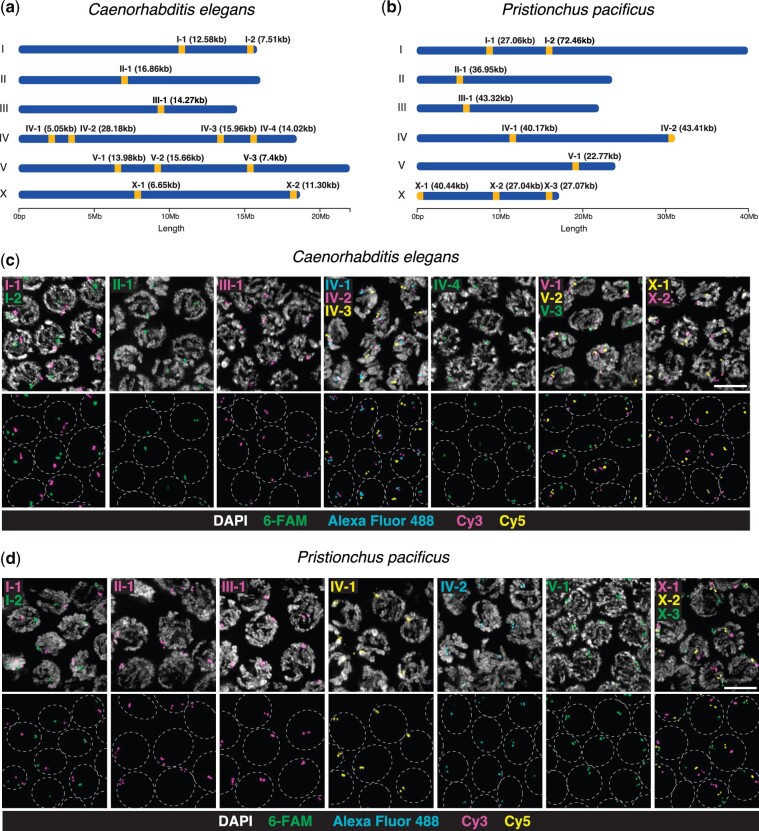
Locus-specific repetitive sequences and FISH probes for *C. elegans* and *P. pacificus*. Chromosome map of oligonucleotide FISH probes for (a) *C. elegans*, and (b) *P. pacificus*; the span of the tandem repeat targeted by each probe is indicated in parentheses. c, d) Meiotic prophase I nuclei at the pachytene stage after FISH with different combinations of probes. Paired homologous chromosomes display adjacent or overlapping signals for each probe. Gray dashed lines outline the position of the nuclei. All images are maximum intensity projections of deconvolved 3D stacks. Scale bar = 5 μm.

Maps showing the genomic position of each probe (Fig. 1, a and b) were generated using the R package chromoMap (Anand and Rodriguez Lopez 2022).

### FISH and immunofluorescence

Sample preparation and hybridization methods were adapted from prior work on *C. elegans* (Phillips *et al.* 2009) and *P. pacificus* (Rillo-Bohn *et al.* 2021), with minor modifications. Fixed tissue was stained in polypropylene tubes rather than on slides. Briefly, age-matched adult hermaphrodites from either *C. elegans* (N2 and Hawaiian CB4856 strains) or *P. pacificus* (PS312 strain) were transferred to a drop of 30 μl of egg buffer (EB) containing 0.05% tetramisole and 0.1% Tween-20 on a coverslip. The gonads were dissected and fixed for 4 min by addition of formaldehyde in EB to 2% final, then transferred to a 1.5-ml tube containing 1 ml PBST. After all samples were dissected and fixed, PBST was replaced with 1 ml methanol prechilled to -30°C and incubated at room temperature for 5 min. Dissected worms were then washed twice with 2× SSCT (0.3 M NaCl, 0.03 M Na citrate, pH 7, 0.1% Tween-20) for 5 min and incubated in 200 μl 2× SSCT containing 50% formamide solution for 4 h or overnight at 37°C. Next, the worms were transferred to a 0.2-ml PCR tube, excess solution was removed from the tissue, and 40 μl of hybridization mix containing 10–250 ng of each probe in hybridization buffer (3× SSC, 48% formamide, 10.6% dextran sulfate) was added. Chromosomal DNA was denatured by incubation in a thermocycler at 91°C for 2 min, followed by overnight hybridization at 37°C in the dark. After this incubation, 100 μl of 2× SSCT was added to each tube, samples were transferred to a 1.5-ml tube, and the tissue was washed 3 times with 2× SSCT for 5 min. After the final wash, the solution was removed and 40 μl of SlowFade Diamond Antifade Mountant with DAPI (Invitrogen) was added. The tissue was transferred to a slide in a minimal volume of mounting medium, overlaid with a #1.5 high-performance coverslip (Zeiss), and sealed with clear nail polish.

For sequential FISH and immunofluorescence, *C. elegans* and *P. pacificus* expressing endogenously tagged SYP-4::HA proteins [*syp-4(ie29)* (Köhler *et al.* 2020) and *syp-4(ie1002)* (Rillo-Bohn *et al.* 2021), respectively] were used to visualize the synaptonemal complex. FISH was performed as described above through the second wash after hybridization. The buffer was replaced with 1× Blocking Reagent (Roche) in PBST and the worms were incubated at least 30 min at room temperature. Goat anti-HA (Novus Biologicals #NB600-362) primary antibodies diluted 1:500 into 1× Blocking Reagent (Roche) in PBST were added and incubated overnight at 4°C. Samples were then washed 3 times with PBST for 5 min and incubated with secondary antibodies [1:500 Donkey antigoat IgG (H + L) conjugated with Cy5 or Cy3, Jackson ImmunoResearch Laboratories, West Grove, PA] for 2 h in the dark at room temperature. Finally, the samples were washed 3 times with PBST for 5 min and mounted as described above.

### Microscopy

The 3D Images were acquired as stacks of optical sections at 0.2 μm z-spacing using a DeltaVision Elite microscope (GE) with an Olympus 100× NA 1.45 objective. All data were deconvolved using the constrained iterative algorithm included with the softWoRx package (GE) using 10 cycles and default settings. Maximum intensity projections were generated from image stacks and then pseudocolored using Fiji software (Schindelin *et al.* 2012).

### Image analysis

The effect of probe concentration on FISH signal intensity was analyzed using a Cy3-labeled probe against *P. pacificus* Chr X-1 at 3 different concentrations, 0.25 ng/μl [36.5 nM], 1.25 ng/μl [182.5 nM], and 6.25 ng/μl [912.3 nM]. The samples were prepared in parallel and imaging conditions and exposure time were the same in each case. Maximum intensity projections of raw images of the Cy3 channel were used for measurements using Fiji (Schindelin *et al.* 2012). The images correspond to the pachytene region of the gonad. A mean background pixel intensity inside the gonad was estimated from the measurement of 3 ROIs that did not contain FISH signals. This value was subtracted from the images to correct for background. FISH signals were then segmented automatically using the Maximum Entropy thresholding algorithm, which we found empirically to identify fluorescent foci with high accuracy and reproducibility. The mean pixel intensity was measured for each signal and used for subsequent analysis. Statistical analysis and graphing were done using GraphPad Prism (v.9.3.0) software.

## Results

### Identification of tandem repeats and design of locus-specific probes for *C. elegans* and *P. pacificus*

Our initial analysis of chromosome-level genome assemblies of *C. elegans* and *P. pacificus* using the Tandem Repeat Finder software (Benson 1999) yielded a large number of repeats: 39,505 and 40,651, respectively. After filtering out those with a total span of less than 5 kb, we analyzed the remaining candidates using BLASTN to confirm a single major cluster in the reference genome (Tables 1 and 2, Fig. 1, a and b). This yielded 1–4 candidate targets per chromosome for each species.

Thirteen probes for *C. elegans* were tested experimentally. The oligonucleotides used as probes ranged from 19 to 30 bases, and targeted repeats spanning 5.1–28.2 kb (Table 1, Fig. 1a). Both tandem repeats on the X chromosome, one near the center and one near the right tip, were previously identified and used successfully as FISH targets, referred as “X center” and “XR” probes, respectively (Phillips *et al.* 2005), while all autosomal targets are described here for the first time. Four additional probes targeting repeats on chromosomes I, II, III, and X showed a smaller unexpected secondary signal during a preliminary FISH test and were excluded from further analysis (Supplementary Table 3). For *P. pacificus*, 10 probes were designed and tested; 2 of these were used to analyze meiotic homolog pairing and synapsis in our recent study (Rillo-Bohn *et al.* 2021). Oligonucleotide probes ranged from 17 to 30 bases, with a target repeat span between 22.8 and 72.5 kb (Table 2, Fig. 1b). None of the repeats we identified are conserved between the 2 species.

### Probe validation

The performance of the probes for *C. elegans* (N2 strain) and *P. pacificus* (PS312 strain) was primarily analyzed in dissected gonads of adult hermaphrodites, which are densely populated with proliferating germline stem cells and nuclei that span the full range of meiotic stages. At meiotic entry, chromosomes undergo a dramatic morphological transition to form elongated territories as they pair with their homologs. At pachytene, when meiotic chromosomes are fully aligned, most nuclei showed 2 adjacent but discrete signals for each FISH probe, corresponding to paired homologous loci separated by the synaptonemal complex. Some nuclei displayed up to 4 smaller signals (Fig. 1,c and d), likely reflecting separation of the 2 sister chromatids of each homolog. All of the probes described here produced reliable signals, and none showed unexpected or nonspecific hybridization to secondary loci. Given this very high success rate, additional probes could likely be designed using more relaxed criteria, particularly regarding the total copy number and genomic span of target sequences. Regarding the hybridization conditions, we found that all the probes produced strong signals at a standard annealing temperature of 37°C, although the predicted T_m_ for some of these sequences deviated quite widely in both directions from this temperature. Together with a body of other empirical experience, we feel that optimization of the predicted T_m_ for repetitive probes is not an important factor in probe design.

Simultaneous detection of multiple targets in the same sample can be accomplished by combining probes with different fluorescent or hapten labels. We performed double- and triple-labeled hybridizations with sets of probes from the same chromosome (Fig. 1c). In *P. pacificus*, 3 widely spaced loci on the X chromosome include one from the left end (X-1), one centrally located (X-2), and one near the right end (X-3), their collinear appearance reveals the orientation of the paired chromosomes at the pachytene stage (Fig. 1d).

For longer target sequences (i.e. *C. elegans* IV-3 and V-1, and *P. pacificus* X-2), we compared the performance of probes comprising 2 nonoverlapping oligonucleotides to hybridization with only one of the oligos; surprisingly, the signal intensities with a single probe were quantitatively indistinguishable from data obtained using 2 labeled oligonucleotides (data not shown).

We also tested 2 *C. elegans* probes for chromosome I (I-1 and I-2) in the divergent Hawaiian (CB4856) strain. Interestingly, while probe I-1 showed reliable signals in this strain, probe I-2 was not detected (data not shown). This likely reflects strain-specific copy number variation of this repeat; other repeats may also show strain-specific differences and probes should thus be tested before use in strains other than N2/VC2010.

### FISH can be combined with immunolocalization in various tissues

**Fig. 2.**
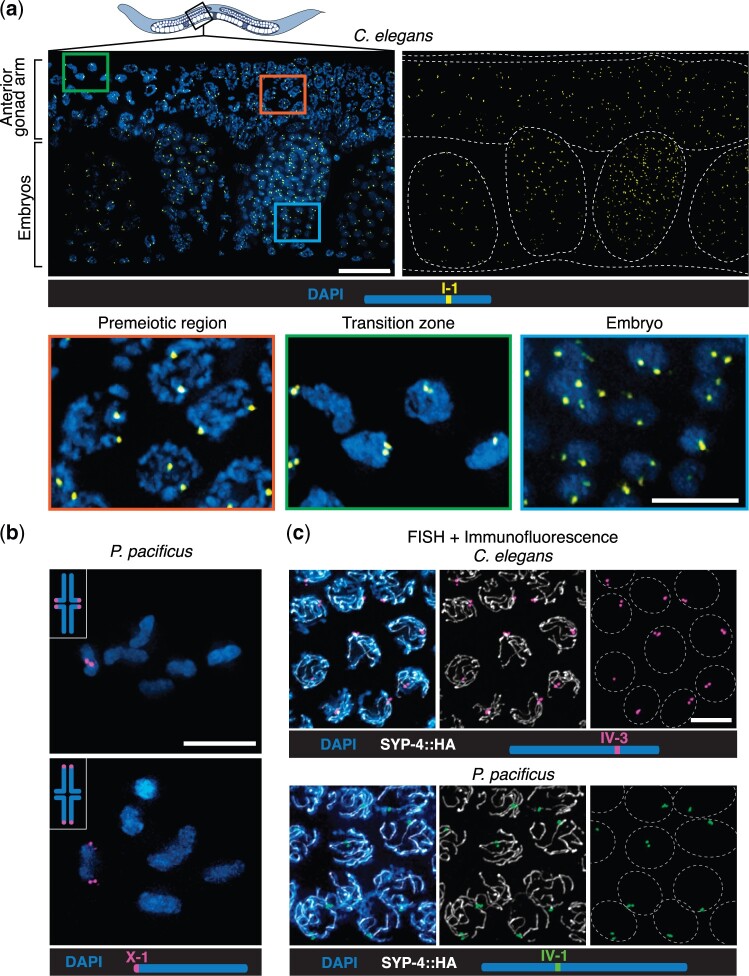
Oligonucleotide FISH probes show robust signals in different tissues and can be combined with immunolocalization. a) Diagram of an adult *C. elegans* hermaphrodite and detail of mid anterior region of the body after FISH with chromosome I-1 probe. The distal anterior gonad arm (top) and 4 embryos (bottom) show consistent hybridization signals. White dashed lines demarcate the worm body, the gonad arm, and the embryos. Scale bar = 15 μm. Higher magnification of nuclei in the premeiotic region with unpaired chromosomes and 2 distant FISH signals (left), in the transition zone after chromosome pairing (center), and in an embryo (right). Scale bar = 5 μm. b) *P. pacificus* diakinesis nuclei showing 6 bivalents after FISH with chromosome X-1 probe, which reveals chiasma position proximal (top) and distal (bottom) to the left of the X chromosome end. Scale bar = 5 μm. c) FISH followed by immunolocalization of the synaptonemal complex protein SYP-4 in strains of *C. elegans* and *P. pacificus* expressing SYP-4::HA. Scale bar = 5 μm. Worm diagram adapted from Wikimedia Commons *C. elegans hermaphrodite adult-en.svg* by K. D. Schroeder, CC-BY-SA 3.0. All images are maximum intensity projections of deconvolved 3D stacks.

To explore the versatility of the set of probes, we assessed the quality of hybridization in different regions of the gonad and in other tissues of the worms using the same FISH protocol. In the premeiotic region of the *C. elegans* gonad, unpaired loci on homologous chromosomes resulted in 2 distinct signals, which became closely associated in transition zone nuclei due to chromosome pairing (Fig. 2a). Two signals per probe were consistently detected in interphase nuclei of embryos at different developmental stages (Fig. 2a).

In *C. elegans* and *P. pacificus*, the position of the single crossover between each pair of homologous chromosomes stochastically determines their orientation during the first meiotic division; the end farther from the crossover site usually separates from its homolog earlier but retains sister chromatid cohesion and leads toward the spindle pole during the first division (Albertson and Thomson 1993; Rillo-Bohn *et al.* 2021). This specification of a “long arm” and a “short arm” along each bivalent is mediated by asymmetric disassembly of axis and synaptonemal complex proteins during diplotene-diakinesis (Martinez-Perez *et al.* 2008; Rillo-Bohn *et al.* 2021). This can be demonstrated using FISH probes that detect distal chromosome regions. For example, hybridization to *P. pacificus* gonads with the X-1 probe reveals that this locus can be on either the long arm or the short arm at diakinesis (Fig. 2b; see also Albertson and Thomson 1993, Rillo-Bohn *et al.* 2021).

In situ hybridization to repetitive targets is advantageous for experiments involving localization of both DNA and proteins because it is relatively tolerant of different fixation methods, allowing optimization of immunofluorescence. We sequentially combined the FISH protocol with immunolocalization of the synaptonemal complex protein SYP-4 in strains of both species expressing HA-tagged SYP-4. This combined protocol resulted in well-preserved morphology and high signal/background for both targets (Fig. 2c). For antigens that are highly sensitive to overfixation, immunolocalization can be performed after an initial light fixation, followed by postfixation to further stabilize the tissue before hybridization (Phillips *et al.* 2009).

### Oligonucleotide probes show consistent hybridization signals over a wide range of concentrations

**Fig. 3.**
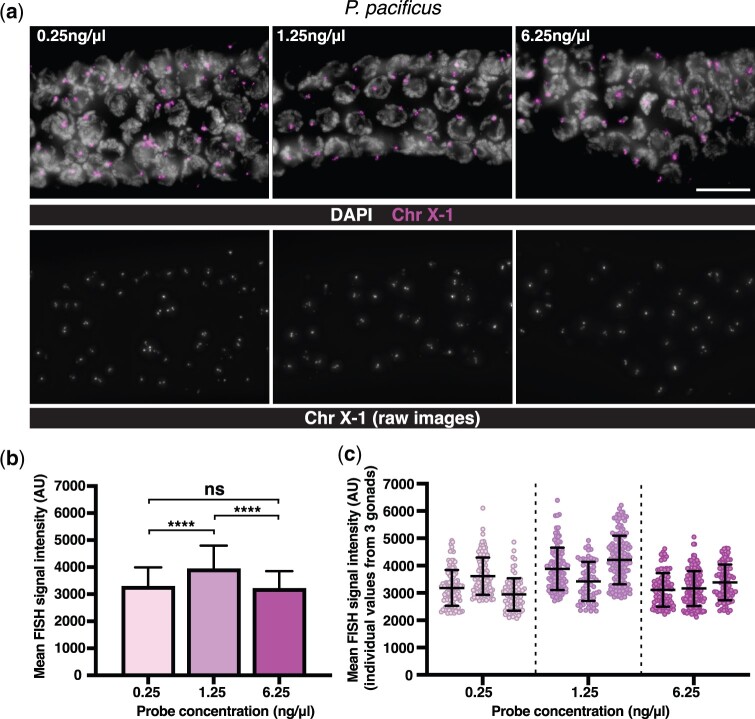
Comparison of signal intensities resulting from hybridization with different probe concentrations. a) Maximum intensity projections of raw (not deconvolved) 3D data showing the pachytene region of *P. pacificus* gonads after FISH with Cy3-labeled X-1 probes at [0.25 ng/μl (36.5 nM), 1.25 ng/μl (182.5 nM), and 6.25 ng/μl (912.3 nM)]. Representative images of Cy3 channel used for image analysis. Scale bar = 10 μm. b) Graph showing the background-corrected FISH signal intensity for each concentration of X-1 probe (mean + SD, ****P < 0.0001 by Welch’s *t*-test). c) Disaggregated data from the 3 images measured per concentration, showing the interspecimen variability (mean ± SD).

To determine the amount of probe required for detection of a typical repetitive locus, we tested 3 different concentrations of *P. pacificus* X-1 probe over a 25-fold dilution range (i.e. 0.25, 1.25, and 6.25 ng/μl). All of these conditions resulted in bright and consistent hybridization signals with low background in the gonad (Fig. 3a). After measuring the background-corrected mean pixel intensity of each FISH signal at the pachytene stage, the mean FISH signal intensity using the intermediate concentration (1.25 ng/μl) was significantly higher than the other 2 concentrations. This likely reflects minor variations in conditions other than probe concentration, since no significant difference was detected between the extremes of the range (0.25 and 6.25 ng/μl) (Fig. 3b). Moreover, we observed similar gonad-to-gonad variation within the same sample as between samples (Fig. 3c). This analysis is consistent with our prior experience that hybridization to repetitive targets is fairly insensitive to variations in probe concentration, and that low probe concentrations are sufficient to detect repetitive targets spanning 5 kb or more with robust signal/background ratios.

## Discussion

We describe a simple method to design locus-specific DNA FISH probes targeting tandem repeats in model and nonmodel organisms. The main requirement for this approach is a chromosome-level genome assembly that includes accurate annotation and quantification of repetitive elements. Using this procedure, we validated probes for the nematodes *C. elegans* and *P. pacificus*, including a least 1 marker for each chromosome. These probes show reliable hybridization, and their synthesis as short oligonucleotides (17–30 bases) facilitates diffusion within different fixed tissues, including intact embryos. This protocol can also be combined with immunolocalization or other cytological methods.

To maximize convenience and reproducibility, probes can be commercially synthesized as labeled oligonucleotides. Given that the lowest concentration we tested (0.25 ng/μl, 36.5 nM, or 10 ng in 40 μl of hybridization mix) resulted in signal/background indistinguishable from a 25-fold higher concentration, a small-scale commercial synthesis of a fluorescently labeled probe with HPLC purification, yielding ∼2 nmol of product, provides sufficient material for at least 2,000 hybridizations. The methods presented here can be easily adapted to generate cost-effective probes in any species with a complete genome assembly based in part on long-read sequencing.

## Data availability

All the software used for this work are publicly available and all the methodological details needed to reproduce this work are included in the manuscript. The sequences of all the oligonucleotide probes tested for *C. elegans* and *P. pacificus* can be found in Tables 1, 2, and Supplementary Table 3.

Supplemental material is available at *G3* online.

## Supplementary Material

jkac121_Supplemental_Tables

jkac121_Supplemental_Note_1
