# Supplementary material for: Robust, versatile DNA FISH probes for chromosome-specific repeats in Caenorhabditis elegans and Pristionchus pacificus
Source: G3 (Bethesda). 2022 May 14;12(7):jkac121. doi: 10.1093/g3journal/jkac121 (PMC9258534; doi:10.1093/g3journal/jkac121)
Supplement: jkac121_Supplemental_Tables [file jkac121_supplemental_tables.docx]

**Supplementary Table 1. Summary of BLASTN results for *C. elegans* probes.**

| **Probe Name** | **Oligonucleotide probe sequences** | **BLASTN Hits (Perfect Hits/Imperfect Hits)** | | | | | |
| --- | --- | --- | --- | --- | --- | --- | --- |
|  |  | **Chr I** | **Chr II** | **Chr III** | **Chr IV** | **Chr V** | **Chr X** |
| I-1 | TCTTTCTGAAATTCTAAGAA | 146/67 | 0/69 | 0/57 | 1/59 | 0/70 | 0/36 |
| I-2 | AATTTTCACTTTCGGTAAAT | 202/31 | 0/15 | 0/21 | 0/12 | 0/29 | 0/23 |
| II-1 | CGAGATGATCGGTCCAGAATACAGC | 0/1 | 87/16 | 0/0 | 0/0 | 0/1 | 0/0 |
| III-1 | CAGTTGAGACTACACCATATACCGG | 0/1 | 0/0 | 95/55 | 0/0 | 0/0 | 0/1 |
| IV-1 | CCGTAAATCTACAGTAATACC | 0/119 | 0/131 | 0/119 | 50/309 | 0/111 | 0/17 |
| IV-2 | TCACTCAAAATCCTGAGCC | 0/12 | 0/8 | 0/10 | 588/504 | 0/7 | 0/3 |
| IV-3 | CTTCTGGTAATGTTCCCATAATTGG | 0/2 | 0/3 | 0/0 | 151/19 | 0/3 | 0/2 |
|  | CTCATAAGTAACTAGTATGGGAC | 0/0 | 0/0 | 0/0 | 152/14 | 0/1 | 0/0 |
| IV-4 | CAGTTCATAAGGGGGACCTT | 0/9 | 0/3 | 0/5 | 240/80 | 0/8 | 0/3 |
| V-1 | CCTCCTGTTTCAGTTTATCATCCT | 01 | 0/4 | 0/6 | 0/6 | 16/123 | 0/6 |
|  | TCTCCTTTTTCAGTTTAGCATCAG | 0/5 | 0/6 | 0/5 | 0/8 | 42/131 | 0/13 |
| V-2 | CTCGTTATGTCGGTTGAAGACACAATTGGA | 0/1 | 0/0 | 0/1 | 0/1 | 244/118 | 0/3 |
| V-3 | GATATCGTAGCGTTTTTTGGTG | 0/7 | 0/4 | 0/7 | 0/10 | 111/82 | 0/7 |
| X-1 | CGCCGGTTTCGCTTTGAGCG | 0/1 | 0/3 | 0/3 | 0/4 | 0/5 | 72/71 |
| X-2 | CACTTCGACTCCATCCACCAGC | 0/2 | 0/4 | 0/4 | 0/6 | 0/7 | 327/33 |
| Probes with extra signals (see Supplementary Table 3) | | | | | | | |
| I | TGTAAGGATTTCCCATTATG | 241/360 | 0/3 | 0/4 | 0/6 | 2/35 | 0/5 |
| II | GCGGAAAAATTGAACCG | 0/32 | 182/225 | 6/46 | 0/48 | 0/54 | 0/31 |
| III | TTGTCTACATAGGGCATCGA | 73/2 | 0/0 | 539/0 | 3/2 | 2/0 | 22/9 |
| X | ATAGCTATGTCGTGTCGTTT | 0/2 | 0/0 | 0/1 | 0/4 | 0/1 | 203/168 |

**Supplementary Table 2. Summary of BLASTN results for *P. pacificus* probes.**

| **Probe Name** | **Oligonucleotide probe sequences** | **BLASTN Hits (Perfect Hits/Imperfect Hits)** | | | | | | | |
| --- | --- | --- | --- | --- | --- | --- | --- | --- | --- |
|  |  | **Chr I** | **Chr II** | **Chr III** | **Chr IV** | **Chr V** | **Chr X** | **pbcontig2** | **pbcontig2855** |
| I-1 | GCCTTGAGCTTCGCCTGTTCTTCGG | 295/133 | 0/1 | 0/3 | 0/1 | 0/1 | 0/0 |  |  |
| I-2 | ACCTCGTGGAGTCCATT | 1571/16 | 4/0 | 1/1 | 13/2 | 4/4 | 8/1 |  | 1/0 |
| II-1 | GGGAGGGTAGACAGTTTACCCACACCAGAA | 0/2 | 374/68 | 0/1 | 0/1 | 0/1 | 0/3 |  |  |
| III-1 | CGTTGACATTGCACGATCGAATTCC | 0/8 | 0/7 | 1087/226 | 0/2 | 1/2 | 0/2 |  |  |
| IV-1 | TCATTGAAATGATCACAATCATTGA | 0/14 | 0/10 | 0/6 | 528/739 | 0/9 | 0/5 | 0/88 |  |
| IV-2 | CTGATGCGTTCTCTACATTTTCGCC | 0/4 | 0/5 | 0/6 | 266/12 | 0/6 | 0/2 |  |  |
| V-1 | GACACTGGCGGTGTTCATTGAGAAC | 0/3 | 0/2 | 0/3 | 0/2 | 218/4 | 0/0 |  |  |
| X-1 | GGTGGTCGACGGCTGCGTCG | 0/4 | 0/1 | 0/2 | 0/2 | 0/1 | 1141/207 |  |  |
| X-2 | TCCGGGGCTTTAGATGAGTTAGA | 0/0 | 0/0 | 0/1 | 0/0 | 0/1 | 169/160 |  |  |
|  | GGAGAAACGATCGAGTTGTATATC | 0/6 | 0/2 | 0/4 | 0/11 | 0/5 | 200/139 |  |  |
| X-3 | TCCCTTTGTTCCGCAGTCCG | 0/9 | 0/7 | 0/5 | 0/2 | 0/4 | 351/454 |  |  |

**Supplementary Table 3.** List of oligonucleotide FISH probes for *C. elegans* with extra signals.

| **Chr** | **Repeat position** | **Span (kb)** | **Oligonucleotide probe sequences** | **Length**  **(b)** | **GC%** | **T_m_** | **Complete tandem repeat motif**  **(probes in bold)** | **Length (b)** |
| --- | --- | --- | --- | --- | --- | --- | --- | --- |
| I | 5273490 - 5290993 | 17.5 | **TGTAAGGATTTCCCATTATG** | 20 | 35 | 25.3 | TCAATACAT**TGTAAGGATTTCCCATTATG** | 29 |
| II | 1459368 - 1479464 | 20.1 | **GCGGAAAAATTGAACCG** | 17 | 47.1 | 26.9 | **GCGGAAAAATTGAACCG** | 17 |
| III | 7632427 - 7683132 | 50.71 | **TTGTCTACATAGGGCATCGA** | 20 | 45 | 31.1 | AAAGCACCCAATATTTAGAGAACAGAAGATTTTGAGAATTACTGCCTCCAGAAATTGATGATTTTCCCATTGAT**TTGTCTACATAGGGCATCGA** | 94 |
| X | 7306206 - 7313838 | 7.63 | **ATAGCTATGTCGTGTCGTTT** | 20 | 40 | 29.8 | **ATAGCTATGTCGTGTCGTTT** | 20 |
