# Supplementary material for: Robust, versatile DNA FISH probes for chromosome-specific repeats in Caenorhabditis elegans and Pristionchus pacificus
Source: G3 (Bethesda). 2022 May 14;12(7):jkac121. doi: 10.1093/g3journal/jkac121 (PMC9258534; doi:10.1093/g3journal/jkac121)
Supplement: jkac121_Supplemental_Note_1 [file jkac121_supplemental_note_1.docx]

**Supplementary Note 1.** Output of Multiple Primer Analyzer (Thermo Fisher) for the set of probes each species.

***C. elegans***

Self-Dimers:

2 dimers for: I-1

5-tctttctgaaattctaagaa->

|||| | | | | ||||

<-aagaatcttaaagtctttct-5

5-tctttctgaaattctaagaa->

|||| ||||

<-aagaatcttaaagtctttct-5

Cross Primer Dimers:

I-2 with III-1

I-2

5-cagttgagactacaccatataccgg->

|| |||||

<-taaatggctttcacttttaa-5

III-1 with V-3

III-1

5-cagttgagactacaccatataccgg->

||||| | | ||

<-gtggttttttgcgatgctatag-5

IV-3a with IV-3b

IV-3a

5-cttctggtaatgttcccataattgg->

||||||| | |

<-cagggtatgatcaatgaatactc-5

IV-3a with IV-4

IV-3a

5-cttctggtaatgttcccataattgg->

| | |||| ||

<-ttccagggggaatacttgac-5

***P. Pacificus***

Self-Dimers:

1 dimer for: II-1

5-gggagggtagacagtttacccacaccagaa->

| | ||||| || || ||||| | |

<-aagaccacacccatttgacagatgggaggg-5

1 dimer for: III-1

5-cgttgacattgcacgatcgaattcc->

||||||

<-ccttaagctagcacgttacagttgc-5

1 dimer for: IV-1

5-tcattgaaatgatcacaatcattga->

|||| ||||

<-agttactaacactagtaaagttact-5

1 dimer for: V-1

5-gacactggcggtgttcattgagaac->

|||| | | ||||

<-caagagttacttgtggcggtcacag-5

Cross Primer Dimers:

IV-1 with V-1

IV-1

5-tcattgaaatgatcacaatcattga->

| || |||| |

<-caagagttacttgtggcggtcacag-5

IV-2 with V-1

IV-2

5-ctgatgcgttctctacattttcgcc->

|||||| | | |

<-caagagttacttgtggcggtcacag-5
